# Cryopreserved exosomes derived from hypoxic Wharton’s jelly mesenchymal stromal cells enhance fibroblast proliferation, upregulate COL1A2 expression, and mitigate senescence

**DOI:** 10.3389/fmed.2025.1692585

**Published:** 2025-12-17

**Authors:** Carolina Quintero-Gil, Wendy Jaraba-Alvarez, Juan P Franco-Salazar, Yurany Blanquiceth, Ferley A Bedoya-Guzmán, Sofía Ortiz-Tamayo, Natalia Quiroz-Correa, Vanesa Sanchez-Giraldo, Karolynn Halpert, Héctor Ortega-Arellano

**Affiliations:** 1 BioXtech, Medellín, Colombia; 2 BioXcellerator, Medellín, Colombia

**Keywords:** exosomes, mesenchymal stromal cells, hypoxic preconditioning, fibroblast proliferation, COL1A2 expression, cellular senescence, regenerative medicine

## Abstract

Mesenchymal stromal cells (MSCs) and their exosomes support regeneration by modulating inflammation and promoting tissue repair. Hypoxic preconditioning enhances the regenerative potential of MSCs by enriching their secretome with trophic and immunomodulatory factors. This study evaluated the biological effects of cryopreserved exosomes from hypoxia-conditioned Wharton’s Jelly MSCs (WJ-MSCs) on human dermal fibroblasts. WJ-MSCs were cultured under hypoxic conditions (5% O₂, 5% CO₂), and exosomes were isolated, characterized and cryopreserved. The initial preparation contained 1.5 × 10^9^ exosomes/mL (~350 μg/mL of total protein). Fibroblasts were treated for 72 h with three exosome dilutions 1:90 (~4 ng/μL), 1:30 (~12 ng/μL), 1:10 (~30 ng/μL), and their effects on proliferation (fluorescence-based cell division assay), COL1A2 gene expression (qPCR), and senescence (β-galactosidase staining) were assessed. Exosome treatment promoted fibroblast proliferation in a dose-dependent manner, with significant effects observed at the 1:30 and 1:10 dilutions (*p* < 0.05 and *p* < 0.01, respectively). COL1A2 expression was significantly upregulated at the 1:30 (*p* = 0.0418), and 1:10 dilutions (*p* = 0.0002), while the 1:90 dilution showed no difference compared to control, indicating that higher exosome concentrations are more effective for extracellular matrix remodeling. Furthermore, the 1:30 and 1:10 dilution significantly reduced β-galactosidase activity in senescent fibroblasts with the 1/10 dilution showing the strongest reduction (*p* = 0.0010), suggesting a potential anti-senescent effect. These findings support hypoxia-conditioned, cryopreserved WJ-MSC exosomes as scalable agents for regenerative therapies. Comparative studies with fresh, frozen, and lyophilized preparations will be essential to clarify storage effects and optimize preservation for clinical use.

## 1 Introduction

Mesenchymal stromal cells (MSCs) have emerged as a cornerstone of regenerative medicine due to their capacity to secrete bioactive factors that modulate inflammation, promote tissue repair, and support overall regeneration (1). In contrast to traditional cell-based therapies, exosomes, nano-sized vesicles secreted by MSCs, present unique benefits such as reduced immune response and effective transport of therapeutic agents (2, 3). These properties position exosomes as ideal candidates for developing advanced therapeutic strategies.

Recent investigations have underscored the potential of hypoxic preconditioning to enhance MSC function by mimicking the cells’ native microenvironment (4). Under hypoxic conditions, MSCs undergo significant metabolic adaptations, including shifts in mitochondrial function, glycolysis, and oxidative phosphorylation (5). These changes not only improve cellular survival but also enrich the secretome with a unique profile of bioactive molecules (6). In particular, exosomes derived from hypoxia-conditioned MSCs appear to encapsulate enhanced therapeutic factors, which may further potentiate their regenerative capacity (7).

In this study, we investigate the regenerative potential of cryopreserved exosomes derived from cryopreserved hypoxia-conditioned Wharton’s Jelly Mesenchymal Stromal Cells (WJ-MSCs) and their impact on fibroblast function. Given that exosomes offer advantages over traditional cell therapies, such as lower immunogenicity and efficient bioactive molecule delivery, their optimization for therapeutic applications remains a key area of interest. Key cellular processes essential for tissue homeostasis, such as fibroblast proliferation, extracellular matrix remodeling, and senescence, remain to be fully understood in the context of cryopreserved exosomes from cryopreserved hypoxia-conditioned WJ-MSCs. Comprehensive characterization of these effects is critical to inform the development of standardized protocols and enhance the therapeutic applicability of exosome-based interventions.

To address this, we evaluated the capacity of exosomes derived from hypoxia-conditioned WJ-MSCs to promote fibroblast proliferation, upregulate COL1A2 expression, a key marker of extracellular matrix remodeling, and attenuate cellular senescence. By integrating insights from cellular metabolism with emerging translational evidence, this study aims to advance our understanding of how hypoxic preconditioning enhances exosome functionality. In addition, we assessed the dose-dependent effects of exosome treatment, identifying an optimal concentration range for therapeutic application. Collectively, these findings may contribute to the refinement of MSC-based regenerative strategies and support the development of interventions aimed at improving tissue repair and mitigating cellular aging in pathological contexts.

## 2 Methodology

### 2.1 MSC culture

Wharton’s Jelly-derived mesenchymal stromal cells (WJ-MSCs) were isolated from human umbilical cords obtained from healthy donors under IRB-approved informed consent, collected between April 2023 and April 2024. MSCs were characterized according to the minimal criteria defined by the International Society for Cell and Gene Therapy (ISCT), including adherence to plastic, expression of CD73, CD90, and CD105, and lack of expression of CD14, CD19, CD34, CD45, and HLA-DR (Beckman Coulter, Inc. DURAClone SC Mesenchymal Stromal Cell. Ref. C34369), along with demonstrated trilineage differentiation potential (Gibco, αMEM, D-MEM/F-12 and supplemented with R&D Systromals®, Catalog # CCM007, R&D Systromals®, Human MSC Functional Identification Kit, Catalog # SC006).

Cells were cultured under a proprietary protocol using Dulbecco’s Modified Eagle Medium (DMEM) low glucose, supplemented with 10% xeno-free human platelet lysate (hPL), under either normoxic (21% O₂, 5% CO₂) or hypoxic (5% O₂, 5% CO₂) conditions at 37 °C. MSCs were expanded between passages 5 and 7 and used for experiments upon reaching 75–85% confluence.

All MSC and exosome batches underwent rigorous sterility controls, including testing for endotoxins, mycoplasma and microbial contamination, to ensure suitability for downstream applications.

### 2.2 Exosome production

#### 2.2.1 Conditioned media obtention

To obtain exosome, and extracellular vesicles (EVs), rich conditioned media from WJ-MSC cultures, a selection process was first conducted to identify suitable cell batches. Cultures reaching 75–85% confluence were chosen to ensure optimal vesicle yield while preserving cell viability. To stimulate exosome release while minimizing hPL-derived contaminants, cells were subjected to a 24-h hPL deprivation protocol.

Multi-layer, single-use cell culture systems (biofactories) were first rinsed twice with Dulbecco’s Phosphate Buffered Saline (DPBS) to remove residual proteins and debris. Subsequently, serum-free, phenol red–free DMEM (low glucose) was added to each biofactory. Cultures were then incubated for 24 h under standard growth conditions (37 °C, 5% CO₂, and 5% O₂ for hypoxic cultures). After the incubation period, conditioned medium was carefully harvested under sterile conditions, transferred into sterile, labeled collection bottles, sealed with parafilm, and stored at −80 °C until further exosome isolation.

#### 2.2.2 Exosome purification

##### 2.2.2.1 Tangential flow filtration

The conditioned medium was centrifuged (800 × g; 30 min) to remove dead cells and large cellular debris. Exosomes were isolated using a Pellicon® Single-Pass Tangential Flow Filtration (TFF) tangential flow filtration. The cell culture medium was filtered using sterile polyethersulfone hollow fiber membranes with a molecular weight cut-off of 10 kDa. The membranes were first washed with three times the volume of sterile PBS (pH 7.4), and the biological samples were subsequently processed using and internal standardized protocol. The exosomes were concentrated 30-fold and diafiltered 10 times in saline solution. The final exosome solution in saline was maintained at a 30X concentration.

##### 2.2.2,2 Size exclusion chromatography

Ten milliliter of exosomes isolated by TFF were applied to a size exclusion chromatography (SEC) column pre-equilibrated with 10 mL of 20 mM HEPES buffer (pH 7.4) to separate exosomes from other molecules (qEV original, 70 nm+; Izon Science Limited). The purified exosomes were then resuspended in a saline solution and cryopreserved at −80 °C.

### 2.3 Exosomes characterization

#### 2.3.1 Nanoparticle tracking analysis

Exosome concentration and size were assessed by Nanoparticle Tracking Analysis (NTA) (NanoSight NS300, Malvern Panalytical) with an integrated CMOS camera. Samples were homogenized, filtered (0.22 μm), and diluted in sterile particle-free saline to 20–100 particles/frame. Final dilutions were recorded to recalculate original concentrations. Measurements were taken at 25 °C (viscosity 0.89–1.00 cP) in static mode with a syringe pump. For each pre-diluted sample (1:100 and 1:1000), three 60-s videos were acquired with constant camera settings. Analysis used consistent software parameters (≥5 frames/track), reporting mean/median diameters, particle concentration (particles/mL), and coefficient of variation across replicates.

#### 2.3.2 Flow cytometry for marker determination

Exosomes were characterized by flow cytometry (CytoFLEX S, Beckman Coulter) using Kaluza software. Vesicles were incubated 30 min at room temperature in the dark with PE-anti-CD9, FITC-anti-CD63, and APC-anti-CD81 (Beckman Coulter) without washing to prevent loss. Isotype-matched controls and 100–500 nm calibration beads set voltages and gates. At least 50,000 events/sample were acquired. Data analysis applied automatic spectral compensation and sequential gating, reporting marker-positive percentages and mean fluorescence intensity (MFI).

#### 2.3.3 Electron microscopy

Exosomes were fixed in 4% paraformaldehyde (1:1) at room temperature for 2 min, 2 μL were placed on carbon–formvar coated copper grids (300 mesh), and dried. Water (2 μL) was added and dried again. Then, 2% aqueous uranyl acetate (2 μL) was applied to the grid for 2 min and dried. The grids were examined under a Transmission Electron Microscope (Tecnai F20 Super Twin TMP) equipped with a field emission source, offering a resolution of 0.1 nm at 200 kV and a maximum magnification of 1.0 MX in TEM mode. Analysis was performed using a STROMAL-FISCHIONE Instruments Model M3000 FP5360/22 HAADF detector operating at 120/200 kV.

#### 2.3.4 Proteomic characterization

Exosomes were lysed in 50 mM Tris (pH 7.5), 100 mM NaCl, and 4% SDS with protease and phosphatase inhibitors, incubated at 95 °C for 10 min, and then lyophilized. Cysteines were reduced and alkylated using TCEP and MMTS, respectively. Tryptic digestion was performed using S-Trap columns (Protifi). Peptides were analyzed by nano HPLC-MS/MS using an Exploris 240 Orbitrap. Separation was carried out on a C18 column (75 μm ID, 50 cm) with a 90-min gradient. The 20 most intense ions (375–1,200 m/z) were selected for MS/MS fragmentation. Data were processed with Proteome Discoverer v2.5 and Mascot, matching MS/MS spectra to the human UniProt-SwissProt database with a decoy set. Peptides were accepted at FDR < 1% (high confidence). Search parameters included fixed cysteine alkylation (MMTS) and variable modifications: N-terminal acetylation, methionine oxidation, and Gln → pyro-Glu. To verify exosomal origin, identified proteins were compared to a curated list of 34 high-confidence exosomal markers (ExoCarta, EVpedia). Detection of ≥ 9–10 markers (≥ 25%) was required to confirm exosomal enrichment.

#### 2.3.5 Protein quantification by Bradford assay

Exosome protein content was quantified using the Pierce™ Coomassie Plus (Bradford) Protein Assay (Thermo Fisher Scientific) following the manufacturer’s instructions. Briefly, exosome samples were diluted (1/10, 1/30, and 1/90) in PBS, and protein concentration was determined by comparison with a bovine serum albumin (BSA) standard curve (0–25 μg/mL). Absorbance was measured at 595 nm using a microplate reader, and values were expressed as ng/μL. Results were calculated from three technical replicates per sample. These protein measurements were used to complement nanoparticle tracking analysis (NTA) data, providing both particle concentration and protein-based quantification metrics for subsequent functional assays.

### 2.4 Functional assays

#### 2.4.1 Thawing and lot reproducibility assessment

Cryopreserved exosome vials were thawed rapidly at 37 °C prior to use. An initial assessment of lot reproducibility and concentration was performed using human fibroblast cultures (Supplementary Figures S2, S3). Exosome concentrations were quantified, with preliminary tests targeting 1.5 billion exosomes/mL corresponding to approximately 350 μg/mL of total protein (quantified by Bradford assay), across volumes ranging from 2 to 5 mL. Variability between lots was evaluated by testing three increasing exosome concentrations, 1:90 (~4 ng/μL), 1:30 (~12 ng/μL), 1:10 (~30 ng/μL) on fibroblast cultures. Following these tests, lots demonstrating minimal variability were pooled to create a mixed exosome preparation for subsequent functional assays. All the subsequent assays were performed as three independent experiments with three technical replicates only for the hypoxic-preconditioned exosomes.

#### 2.4.2 Proliferation assays

For the proliferation assay, fibroblasts (C0135C, Human Dermal Fibroblasts, adult (HDFa), Thermo Fisher Scientific, Inc., Waltham, MA, United States), from P10 to P15, were seeded in a P12 plate with three replicates per condition, seeding 100,000 cells per well. Cells were exposed to exosomes for 48 h. Proliferation was quantified using a fluorescence-based assay employing CellTracker Green (CTG, Item: C7025; Life Technologies, Thermo Fisher Scientific, Inc., Waltham, MA, United States), a dye that dilutes with each cell division, decreasing fluorescence and similar to CFSE (Carboxyfluorescein Succinimidyl Ester). Flow cytometry (BD Accuri™ CSampler) was utilized to measure the reduction in fluorescence intensity, thereby providing a quantitative assessment of the induction of cell division. Data were analyzed on a BD Accuri™ C Program. First, viable fibroblasts were selected by applying a primary gate on forward scatter area (FSC-A) versus side scatter area (SSC-A) to exclude debris and aggregates, retaining only events with the characteristic size and granularity of fibroblasts. Cell viability was then confirmed by exclusion of propidium iodide (PI)-positive events; PI is a DNA-binding dye that stains non-viable cells, thereby allowing removal of dead cells from subsequent analyses. After establishing a homogeneous, viable fibroblast population, CellTracker Green (CTG) fluorescence (FL1 channel) was evaluated against SSC-A. Proliferating cells were identified as the subpopulation that exhibited decreased CTG fluorescence intensity relative to the parent fibroblast population, reflecting dye dilution during successive cell divisions. The percentage of proliferating cells was thus calculated as the fraction of CTG-low events within the gated viable fibroblast population.

#### 2.4.3 Gene expression analysis

To assess extracellular matrix production, COL1A2 mRNA levels were quantified by qPCR. Total RNA was isolated from treated fibroblasts using the Invitrogen™ PureLink™ RNA Mini Kit. Following isolation, samples were treated with the Turbo DNA-free kit (Invitrogen) to remove contaminating genomic DNA, and cDNA synthesis was performed using SuperScript (Invitrogen, AM1907). qPCR was conducted on an ABI 7500 PCR systromal using COL1A2-specific primers (Forward: GGCCCTCAAGGTTTCCAAGG; Reverse: CACCCTGTGGTCCAACAACTC) with PUM1 as the housekeeping gene (primer specifications and cycling conditions provided in Supplementary material). Relative expression levels were calculated using the 2^−^ΔΔCt method, allowing for the quantification of differences between control and treated groups.

#### 2.4.4 Senescence-modulation assay

Human fibroblasts were induced into replicative senescence through serial passaging up to passage 30, approximating the Hayflick limit. Cells were then incubated for 48 h with exosomes at the previously optimized 1:30 dilution. Senescence was quantified by senescence-associated β-galactosidase staining following established protocols. Early-passage fibroblasts served as negative controls, whereas late-passage cells treated with vehicle alone functioned as positive senescence controls. The extent to which exosome treatment reduced the senescent phenotype was calculated relative to these controls.

## 2.5 Statistical analysis

Proteomic analysis was performed using FunRich 3.1.3 for the database comparisons and SRplot for expression analysis. Technical replicates from proliferation, gene expression, and anti-senescence assays were averaged to yield one value per biological replicate (*n* = 3 per condition). Given the sample size, we used a pre-specified non-parametric approach: Kruskal–Wallis for multi-group comparisons and Mann–Whitney U for pairwise tests (two-tailed; exact *p* values reported). Plots display individual biological replicates with median and interquartile range. Statistical analyses were conducted using Prism software (GraphPad Prism), and results with a *p*-value < 0.05 were considered statistically significant.

## 3 Results

### 3.1 Characterization of WJ-MSCs and their exosomes, followed by optimization of exosome concentration

**Figure 1:**
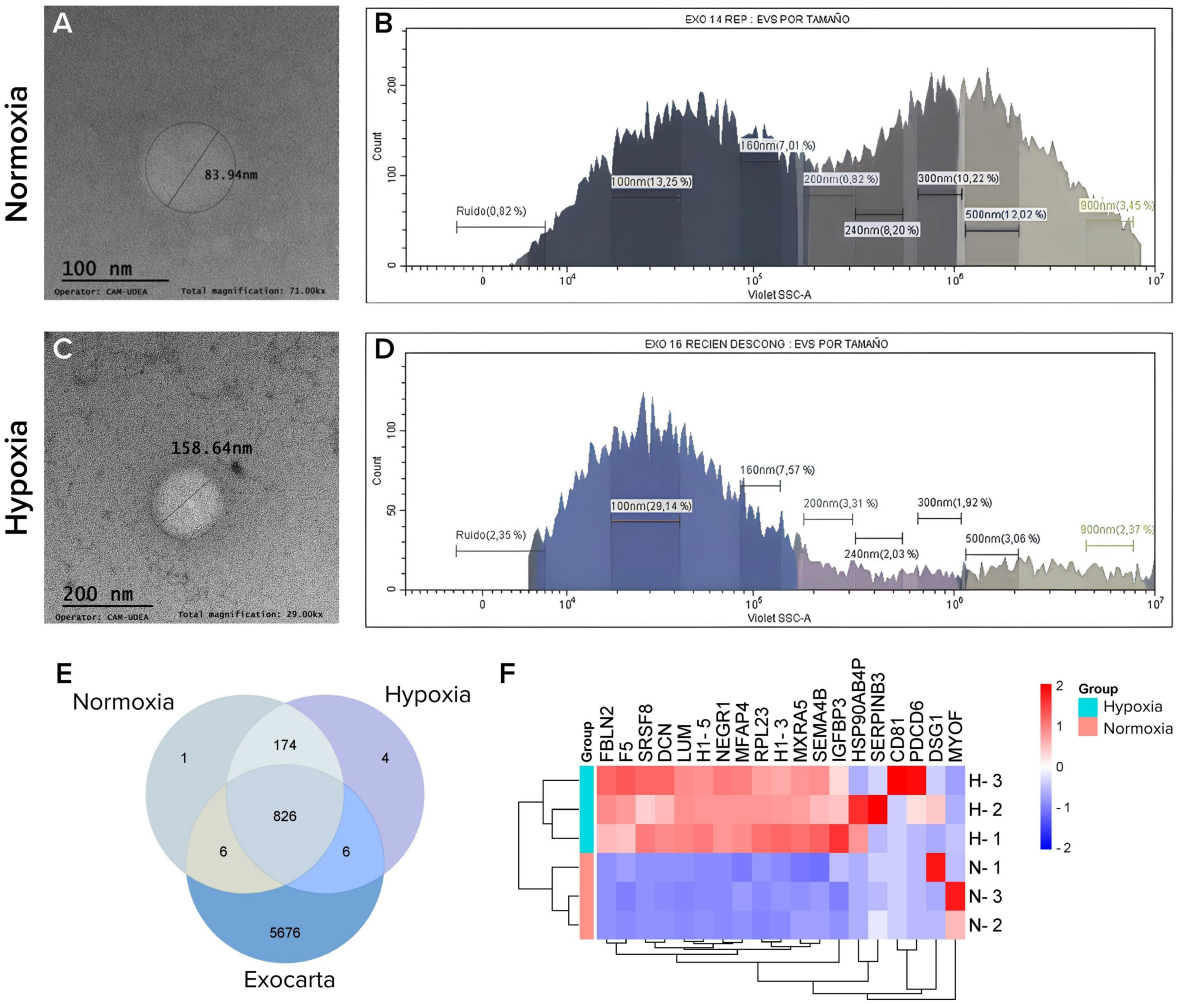
Exosomes characterization. **(A,B)** Exosomes from WJ-MSCs cultured under normoxic conditions, **(C,D)** exosomes from WJ-MSCs cultured under hypoxic conditions. **(A,C)** Images from electronic microscopy, **(B,D)** particle size distribution, **(E)** Venn diagram with the number of proteins identified in normoxic or hypoxic exosomes compared with the Exocarta database, (**F)** heatmap with differentially expressed proteins in normoxia and hypoxia.

In this study, we first isolated and cultured MSCs from the Wharton’s jelly tissue of umbilical cord under two oxygen conditions, and the cells showed typical fibroblast-like, spindle-shaped appearance, and were positive (≥95%) for typical MSC surface markers such as CD73, CD90, and CD105 and negative (≤2.0%) for hematopoietic stromal cell markers (CD34, CD11b, CD15, CD45, HLA DR) (Supplementary Figure S1). Exosomes were characterized using flow cytometry to detect the presence of surface markers CD9, CD63, and CD81. The analysis revealed extracellular vesicle (EV) populations with sizes ranging from 100 to 160 nm, as confirmed by transmission electron microscopy (TEM) (Figures 1A,C). Marker expression analysis showed that the EVs were positive for CD9, CD63, and CD81, supporting the identification of the vesicles as exosomes. The initial exosome concentration from the tested isolated lots was 1.5 billion particles per mL corresponding to approximately 350 μg/mL of total protein (quantified by Bradford assay).

For screening assays assessing proliferation, senescence, and collagen expression, we evaluated three dilution ratios: 1:90 (~4 ng/μL), 1:30 (~12 ng/μL), 1:10 (~30 ng/μL) of the hypoxic-preconditioned exosomes. A 1/10 dilution indicates that the original exosome preparation was diluted tenfold, resulting in a final concentration of 150 million particles per mL; similarly, the 1/30 and 1/90 dilutions correspond to approximately 50 million and 16.7 million particles per mL, respectively. Preliminary tests to determine the optimal concentration range (Supplementary Figure S2) and assess lot variability (Supplementary Figure S3) are presented in the Supplementary material.

### 3.2 Enhanced exosome yield and distinct proteomic profile under hypoxic conditions

Hypoxic preconditioning of MSCs led to a marked increase in exosome yield and a higher degree of exosomal particle enrichment. Specifically, under hypoxic conditions, 76.76% of the exosomes measured between 100 and 160 nm, in contrast to only 42.23% observed under normoxic conditions (Figures 1B,D). This substantial increase in yield underscores the potential of hypoxic conditioning to optimize exosome production.

The Venn diagram qualitative analysis of the proteomic characterization shows that, when comparing proteins identified under Normoxia (Supplementary Table S1) and Hypoxia (Supplementary Table S2) with those reported in the ExoCarta database (Supplementary Table S3, downloaded on July 26, 2024), 174 proteins were common to both experimental conditions but not present in ExoCarta. Additionally, under normoxia, IGHV3-13 was uniquely detected, while CSN3, SCRN1, IGLV3-21, LPA, KRT77, and IQGAP2 were present both in normoxia and exocarta, reflecting mainly immune and secretory proteins. In contrast, hypoxia exclusively contained COL28A1, CSN1S2, HSP90AB4P, and NEGR1, and in combination with exocarta showed CTSL, ARHGAP1, RPL21, PDCD6, SRSF8, and PTGES3, indicating enrichment in stress, cytoskeletal and apoptotic regulators. These patterns suggest a shift from immune/secretory cargo under normoxia to stress- and remodeling-related proteins under hypoxia (Figure 1E).

Proteomic profiling of these hypoxia-conditioned exosomes revealed a distinct molecular signature, with 17 proteins showing differential expression (note that these proteins are mostly different from the found in only normoxia or only hypoxia in the venn diagram), which were mainly associated with: extracellular matrix (FBLN2, DCN, LUM, MFAP4, MXRA5), coagulation and immune response (F5, SERPINB3, HSP90AB4P), transcription and RNA processing (SRSF8, RPL23, HA-5, H1-3), cell adhesion and membrane proteins (NEGR1, SEMA4B, CD81, DSG1), cell death and vesicle trafficking (PDCD6, MYOF). These proteins are critically involved in pathways related to cellular growth regulation, the oxidative stress response, and the maintenance of structural integrity, including processes such as actin and collagen organization (Figure 1F).

### 3.3 Enhanced fibroblast proliferation following exosome treatment

**Figure 2:**
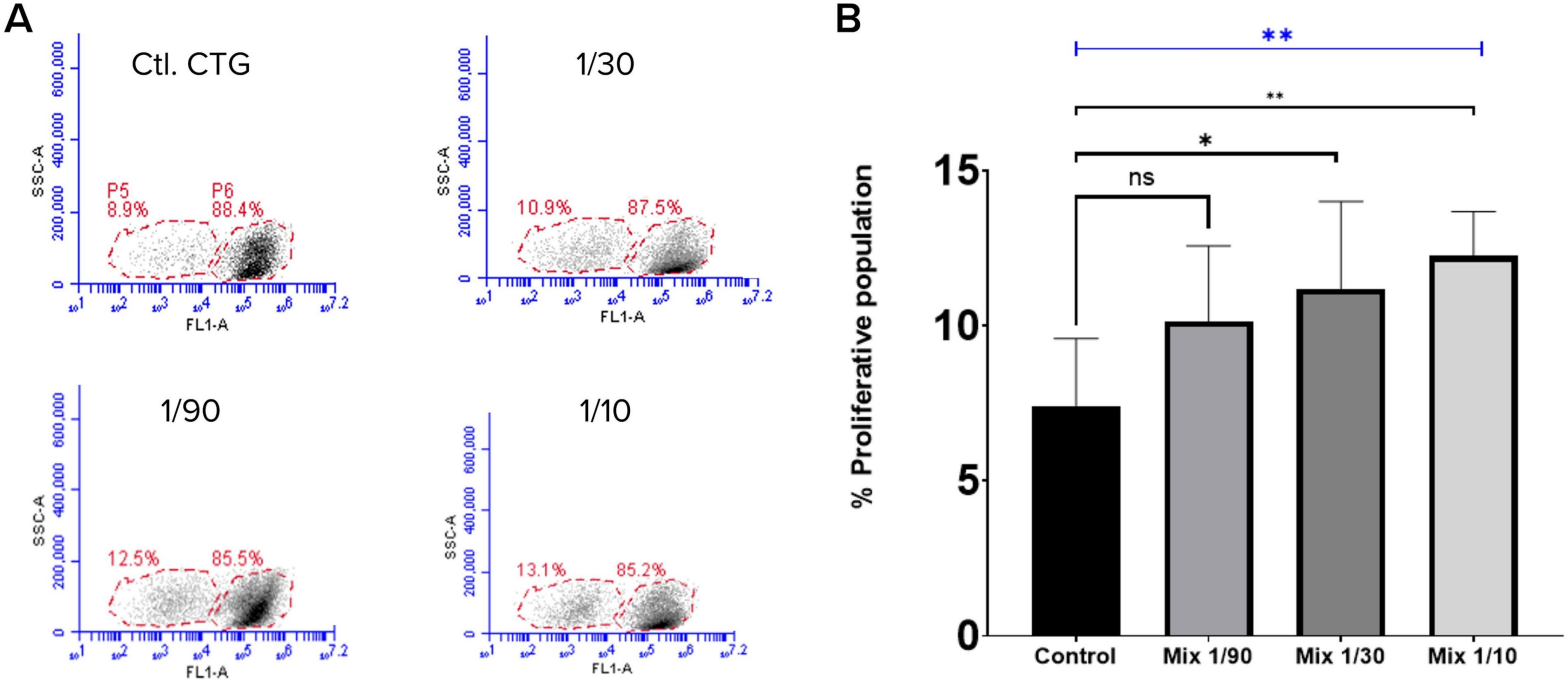
Representative flow cytometry plots and quantitative analysis of fibroblast proliferation. **(A)** Flow cytometry plots showing the gating strategy used to identify proliferating fibroblasts labeled with CellTracker Green (CTG). Forward scatter (FSC-A) versus side scatter (SSC-A) gating was applied to select viable fibroblasts, followed by exclusion of propidium iodide (PI)-positive events to remove dead cells. The remaining homogeneous fibroblast population was analyzed for CTG fluorescence (FL1 channel). The black arrow highlights the population with reduced fluorescence intensity, corresponding to actively dividing cells due to dye dilution. Each panel corresponds to a different treatment condition: Control (Ctl. CTG), Exosome Mix at 1/90, 1/30, and 1/10 dilutions. **(B)** Bar chart quantifying the percentage of proliferative fibroblast populations under each condition. The blue horizontal line represents the Kruskal–Wallis test, confirming a significant overall difference among groups (*p* < 0.01). Black lines indicate pairwise comparisons using the Mann–Whitney test, demonstrating that 1/30 and 1/10 dilutions significantly enhance fibroblast proliferation (*p < 0.05 and **p < 0.01, respectively). These results suggest a dose-dependent response of exosomes in promoting cell proliferation.

Fibroblasts exposed to exosomes for 48 h showed an increased proliferative population, as indicated by flow cytometry (Figure 2A). The 1/30 and 1/10 exosome dilutions significantly enhanced fibroblast proliferation (*p* < 0.05 and *p* < 0.01, respectively), whereas the 1/90 dilution did not differ from the control (ns) (Figure 2B). These findings suggest a dose-dependent effect of exosomes on fibroblast proliferation.

### 3.4 COL1A2 gene expression analysis

**Figure 3:**
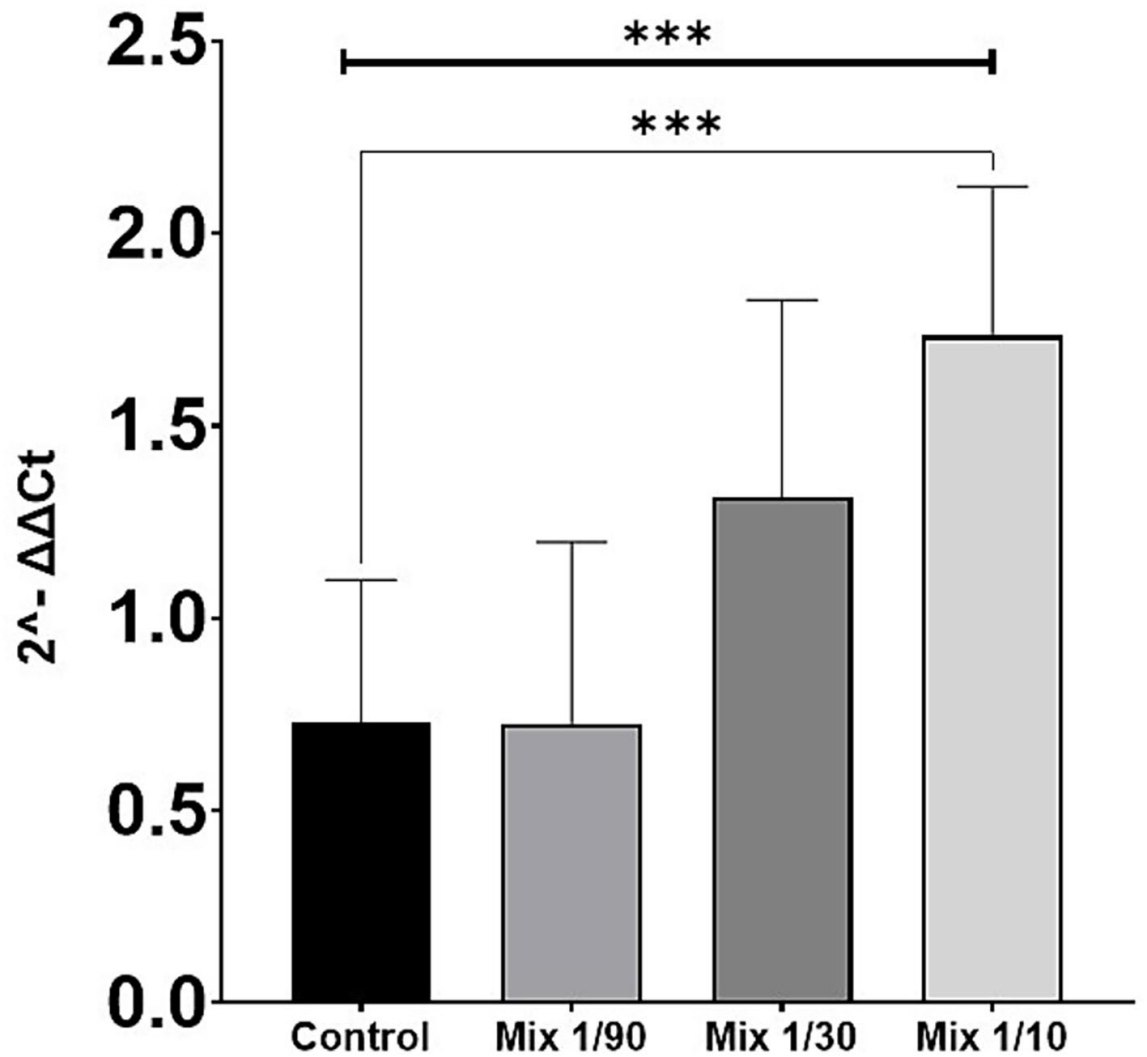
Dose–response analysis of exosome treatment using a mix of lots in fibroblasts (COL1A2 expression). Mixed exosomes from three independent MSC isolation lots (1.5 × 10^9^ exosomes/mL) were tested at dilutions of 1/90, 1/30, and 1/10, compared to control. Expression analysis showed that the 1/90 dilution had levels comparable to control, whereas the 1/30 and 1/10 dilutions induced a marked increase in relative expression (2^−^ΔΔCt). A Kruskal–Wallis test indicated highly significant overall differences among groups (*p* = 0.0007; shown by the top bracket with ***). *Post hoc* Mann–Whitney tests showed no difference for control vs. 1/90 (ns), a significant increase for control vs. 1/30 (*p* = 0.0418; *), and a highly significant increase for control vs. 1/10 (*p* = 0.0002; ***). Thin brackets indicate the pairwise Mann–Whitney comparisons. The assay was performed independently three times, each with three technical replicates per condition.

To evaluate the dose-dependent effects of exosomes derived from WJ-MSCs, mixed exosomes from three independent isolation lots were tested. The concentration used was 1.5 × 10^9^ exosomes/mL, and samples were diluted at ratios of 1/90, 1/30, and 1/10, with untreated cells serving as the control group. Expression analysis revealed that the 1/90 dilution produced levels comparable to the control. In contrast, the 1/30 and 1/10 dilutions resulted in a significant increase in relative gene expression, as measured by the 2^−^ΔΔCt method. These findings demonstrate a clear dose-dependent response, indicating that higher concentrations of exosomes are associated with enhanced biological activity (Figure 3).

### 3.5 Exosome concentration-dependent modulation of senescence in Hayflick-induced fibroblasts

**Figure 4:**
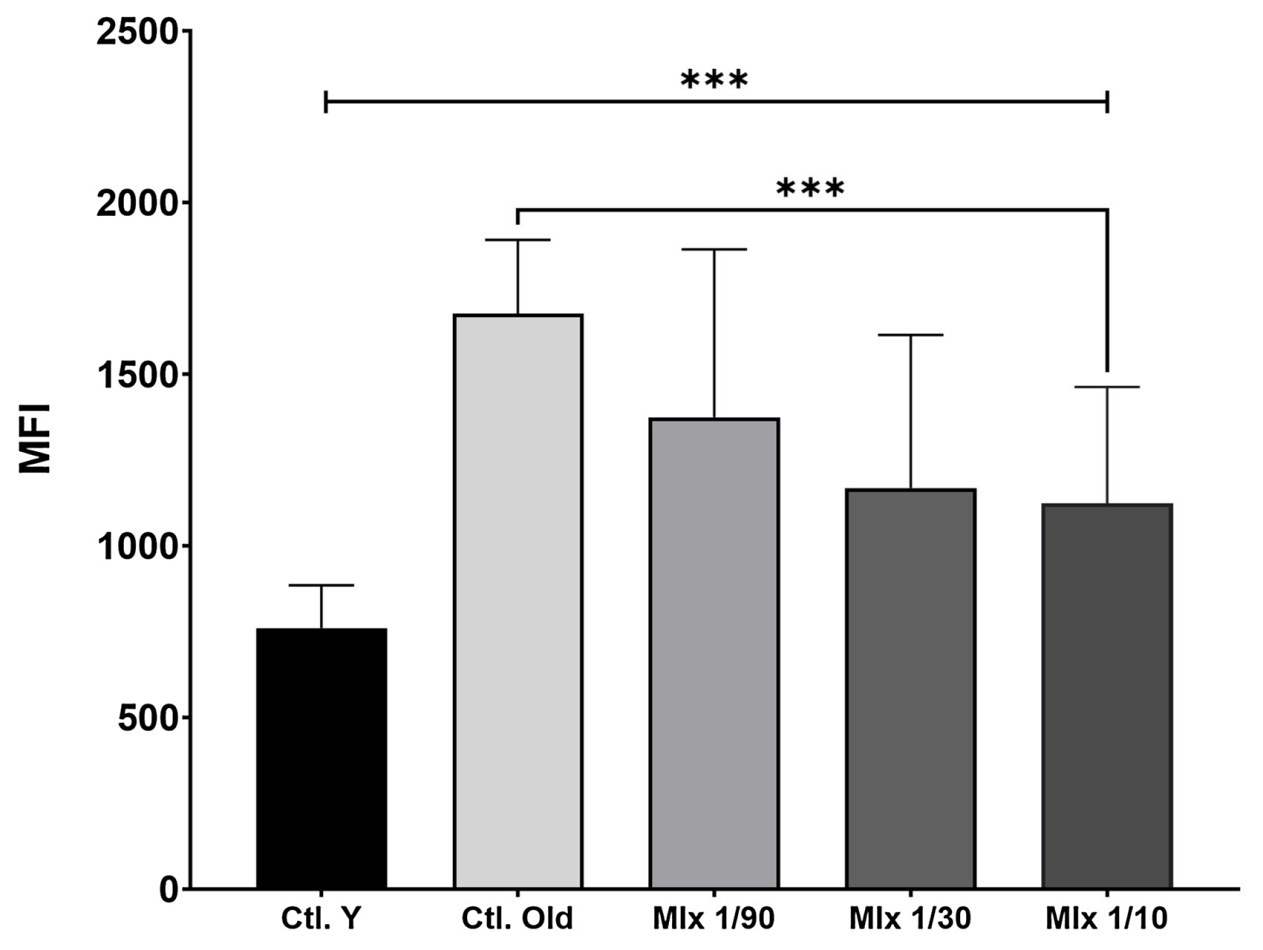
Effect of exosome treatment on β-galactosidase (β-gal) activity in fibroblasts subjected to Hayflick-induced senescence. The outcome is mean fluorescence intensity (MFI) of β-gal staining. “Ctl. Y” denotes young (non-senescent) fibroblasts; “Ctl. Old” denotes senescent controls. As expected, Ctl. Old showed higher MFI than Ctl. Y. Mixed exosomes from three independent MSC isolation lots (1.5 × 10^9^ exosomes/mL, ~350 μg/mL) were tested at dilutions of 1:90 (~4 ng/μL), 1:30 (~12 ng/μL), 1:10 (~30 ng/μL) compared to control. At 1/90, β-gal activity did not differ from Ctl. Old. In contrast, 1/30 and 1/10 reduced β-gal activity relative to Ctl. Old, with the largest reduction at 1/10. Kruskal–Wallis test indicated overall differences (*p* = 0.0010; top bracket). Exact two-tailed Mann–Whitney comparisons showed Ctl. Old vs. 1/90: ns; Ctl. Old vs. 1/30: *p* = 0.0188; Ctl. Old vs. 1/10: *p* = 0.0008***. Thin brackets indicate pairwise Mann–Whitney comparisons. Data are summarized across biological replicates as group medians with interquartile ranges (IQR). The assay was performed independently three times, each with three technical replicates per condition.

Hayflick-induced senescence in fibroblasts was assessed via β-galactosidase (β-gal) activity, estimated by fluorescence intensity. “Ctl. Y” denotes young (non-senescent) fibroblasts, while “Ctl. Old” represents senescent cells. Senescent fibroblasts (Ctl. Old) exhibited a pronounced increase in β-galactosidase activity compared to young fibroblasts, consistent with expected senescence-associated changes. To assess the potential rejuvenating effects of exosomes, treatments were applied using a mixed lot preparation at three dilutions: 1/90, 1/30, and 1/10. At the 1/90 dilution, β-galactosidase activity remained comparable to that of the senescent control group, indicating no significant effect. However, both the 1/30 and 1/10 dilutions resulted in a statistically significant reduction in β-gal activity relative to Ctl. Old, with the 1/10 dilution showing the most pronounced decrease. These findings suggest a dose-dependent effect of exosome treatment on senescence-associated β-galactosidase activity (Figure 4).

## 4 Discussion

The findings from this study underscore the enhanced therapeutic potential of cryopreserved exosomes derived from hypoxia-preconditioned Wharton’s Jelly Mesenchymal Stromal Cells (WJ-MSCs) in promoting fibroblast proliferation, upregulating COL1A2 expression, and mitigating cellular senescence.

Hypoxic preconditioning of MSCs has been shown to augment their regenerative capabilities. This enhancement is primarily attributed to the activation of hypoxia-inducible factors (HIFs), which regulate genes involved in processes such as inflammation, migration, proliferation, differentiation, angiogenesis, metabolism, and apoptosis. Consequently, exosomes secreted under hypoxic conditions are enriched with bioactive molecules that can induce physiological changes in recipient cells (8, 9). In our study, hypoxic preconditioning significantly enhanced the yield and enrichment of exosomes derived from WJ-MSCs. Specifically, 76.76% of exosomes within the 100–160 nm range were produced under hypoxic conditions, compared to 42.23% under normoxia. These results confirmed that hypoxia promotes exosome biogenesis in WJ-MSCs, likely through HIF-1α-mediated pathways that regulate vesicle trafficking and release (10).

Cryopreserved exosomes derived from hypoxia-conditioned WJ-MSCs significantly enhanced fibroblast proliferation in a dose-dependent manner, with the 1/30 and 1/10 dilutions showing the most notable effects. This proliferative response is consistent with the paracrine activity previously described for WJ-MSCs, particularly through the secretion of bioactive molecules that modulate fibroblast behavior. In a study by Kim et al., WJ-MSC-conditioned medium (WJ-MSC-CM) significantly increased the proliferation and migration of normal human skin fibroblasts, while also upregulating genes involved in re-epithelialization (TGF-β2), neovascularization (HIF-1α), and fibroproliferation (PAI-1), ultimately promoting wound healing in a murine model (11). Our findings suggest that exosomes may be key mediators of these effects, and that hypoxic preconditioning enhances their potency by enriching their cargo with regenerative signals. These findings further underscore the therapeutic potential of hypoxia-primed WJ-MSC-derived exosomes in applications that require fibroblast activation, such as chronic wound healing and tissue regeneration. This is in line with previous studies demonstrating that hypoxic conditions enhance MSC proliferation and survival, thereby amplifying their regenerative and paracrine effects (9, 12). Although the *in vitro* findings in fibroblasts suggest potential relevance to wound repair and anti-senescence mechanisms, these implications remain speculative and require validation in *in vivo* and translational models. Moreover, exosomes derived from Wharton’s Jelly MSCs have shown superior biological activity compared to those from adipose-derived MSCs (AD-MSCs), particularly in promoting wound healing. Proteomic analyses revealed that WJ-MSC exosomes are enriched in key proteins such as fibrinogen beta chain (FGB), which are associated with enhanced keratinocyte proliferation and migration—critical processes in tissue repair (13).

Furthermore, proteomic profiling of hypoxia-conditioned cryopreserved exosomes revealed 17 differentially expressed proteins involved in key biological processes such as oxidative stress response, cytoskeletal organization, and extracellular matrix remodeling. Notably, proteins related to actin and collagen dynamics were enriched, suggesting a role in structural tissue repair. This aligns with evidence that WJ-MSC-derived exosomes can modulate fibroblast activity and enhance collagen synthesis (14). In our assays, fibroblasts treated with cryopreserved exosomes exhibited a dose-dependent upregulation of COL1A2 expression, suggesting a positive impact on extracellular matrix remodeling and tissue repair. The 1/90 dilution showed expression levels comparable to the control, while the 1/30 and 1/10 dilutions induced a marked increase in COL1A2 expression, as measured by the 2^−^ΔΔCt method. These findings underscore the importance of optimizing exosome concentration, as higher doses appear to be more effective in promoting matrix-related gene expression.

Additionally, exosome treatment at a 1/10 dilution significantly reduced β-galactosidase activity in senescent fibroblasts, indicating a potential role in mitigating cellular aging. This reduction was dose-dependent, with the 1/10 dilution showing the strongest effect compared to the 1/30 and 1/90 dilutions, the latter of which showed no significant change. These findings suggest that higher concentrations of exosomes may exert anti-senescent effects, which could be particularly valuable in regenerative medicine applications aimed at enhancing tissue repair and delaying cellular aging. These findings are consistent with previous studies showing that mesenchymal stromal cell-derived exosomes (MSC-Exo) can reverse senescence phenotypes by modulating key signaling pathways such as MAPK, AKT, STAT3, and ERK1/2, reducing ROS accumulation, and enhancing SIRT1 expression (15). Importantly, our exosomes were derived from Wharton’s Jelly MSCs (WJ-MSCs), a source that has demonstrated superior regenerative potential compared to other MSC types. For instance, Liao et al. (20) reported that UCMSC-derived exosomes prevented senescence in mouse primary tubular epithelial cells by downregulating senescence markers and SASP factors. Moreover, placental MSC-derived EVs have been shown to inhibit senescence by activating p53 and p21 pathways, and bone marrow MSCs (BMSCs) have demonstrated anti-senescence effects through Wnt/β-catenin signaling, reducing oxidative stress and DNA damage (16). These comparative findings underscore the importance of exosome source selection, with WJ-MSCs emerging as particularly promising candidates for anti-aging and regenerative therapies.

In addition, a growing body of literature supports the notion that exosomes exert broad biological effects through multiple mechanisms. For example, An et al. (17) reviewed the role of adipose-derived stem cell exosomes (ADSCs-Exo) in wound healing and demonstrated their capacity to modulate immune responses, promote angiogenesis, accelerate cellular proliferation and re-epithelialization, and regulate collagen remodeling. These effects are mediated by diverse molecular pathways, including miRNA transfer, cytokine signaling, and extracellular matrix interactions. Although our study did not directly assess wound healing, oxidative stress, or anti-aging pathways, the observed anti-senescent effects align with the multifaceted roles of MSC-derived exosomes described in the literature (17). Therefore, our findings may contribute to the broader understanding of exosome-based therapies and suggest their potential application in regenerative medicine, particularly in contexts where modulation of cellular senescence is relevant.

Prior reports have established the superior regenerative activity of hypoxia-conditioned MSC exosomes, our study specifically evaluated the functionality of cryopreserved hypoxia-derived vesicles, to understand mainly the advantages of hypoxic preconditioning (8). While this approach represents a methodological limitation, since normoxic-derived exosomes were not included in further assays, previous proteomic analyses revealed that hypoxia-conditioned cells exhibit differential expression of proteins associated with cellular stress responses and extracellular matrix remodeling. These molecular features are consistent with the observed effects on fibroblast proliferation and senescence, suggesting that hypoxic conditioning could enhance the therapeutic potency of exosomes. Therefore, we hypothesize that this preconditioning strategy could enrich the bioactive profile of exosomes for regenerative applications.

Another methodological limitation that warrants discussion is the fact that all exosomes in this study were cryopreserved to model real-world translational workflows. Consequently, we did not include fresh exosome controls, which restricts conclusions regarding possible cryopreservation-induced changes. However, it is important to note that in our experimental design, exosomes were not subjected to repeated freeze–thaw cycles, which are known to significantly compromise vesicle integrity and bioactivity, and our results demonstrated preserved biological function, consistent with previous reports which highlight that storage at −80 °C maintains exosome morphology, cargo content, and functional properties when properly handled (18). These findings support the feasibility of cryopreservation for clinical applications. Nevertheless, future studies should explore alternative preservation strategies such as lyophilization or hydrogel encapsulation, which have shown promise in maintaining exosome stability and enabling room-temperature storage (19). Comparative analyses including fresh, frozen, and lyophilized preparations will be essential to fully elucidate the impact of storage conditions on therapeutic efficacy, and to underscore the importance of optimizing preservation methods that support the scalability and reproducibility of exosome-based therapies.

Collectively, these results suggest that hypoxic preconditioning of WJ-MSCs enhances the therapeutic potential of their derived exosomes. These exosomes not only promote fibroblast proliferation but also modulate gene expression related to extracellular matrix production and reduce cellular senescence. These findings provide valuable insights into optimizing MSC culture conditions to develop more effective exosome-based therapies for tissue repair and regeneration.

## Data Availability

The original contributions presented in the study are included in the article/Supplementary material, further inquiries can be directed to the corresponding author.
