# Supplementary material for: Cryopreserved exosomes derived from hypoxic Wharton’s jelly mesenchymal stromal cells enhance fibroblast proliferation, upregulate COL1A2 expression, and mitigate senescence
Source: Front Med (Lausanne). 2025 Dec 17;12:1692585. doi: 10.3389/fmed.2025.1692585 (PMC12753964; doi:10.3389/fmed.2025.1692585)
Supplement: Supplementary file 4 [file Image_1.pdf]

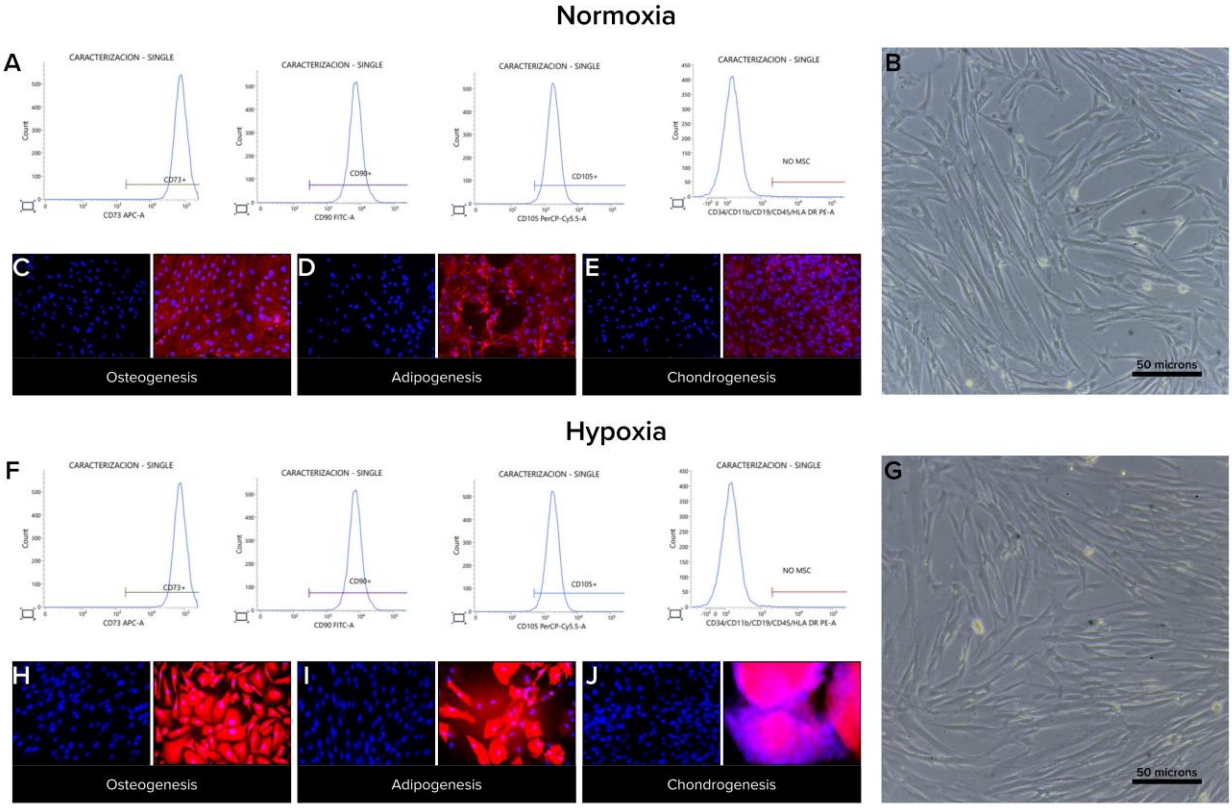

Supplementary Figure 1. WJ-MSCs ISCT minimal criteria characterization. (A-E) WJ-MSCs cultured under normoxic conditions, (F-J) WJ-MSCs cultured under hypoxic conditions. (A and F) Flow cytometry results for the mesenchymal cell markers: CD73, CD90 and CD105; and the NO MSC markers (CD34, CD11b, CD15, CD45, HLA DR). (B and G) morphologic characterization. (C and H) Osteogenic, (D and I) adipogenic and (E and J) chondrogenic differentiation by immunofluorescence.
