# Supplementary material for: Cryopreserved exosomes derived from hypoxic Wharton’s jelly mesenchymal stromal cells enhance fibroblast proliferation, upregulate COL1A2 expression, and mitigate senescence
Source: Front Med (Lausanne). 2025 Dec 17;12:1692585. doi: 10.3389/fmed.2025.1692585 (PMC12753964; doi:10.3389/fmed.2025.1692585)
Supplement: Supplementary file 5 [file Image_2.pdf]

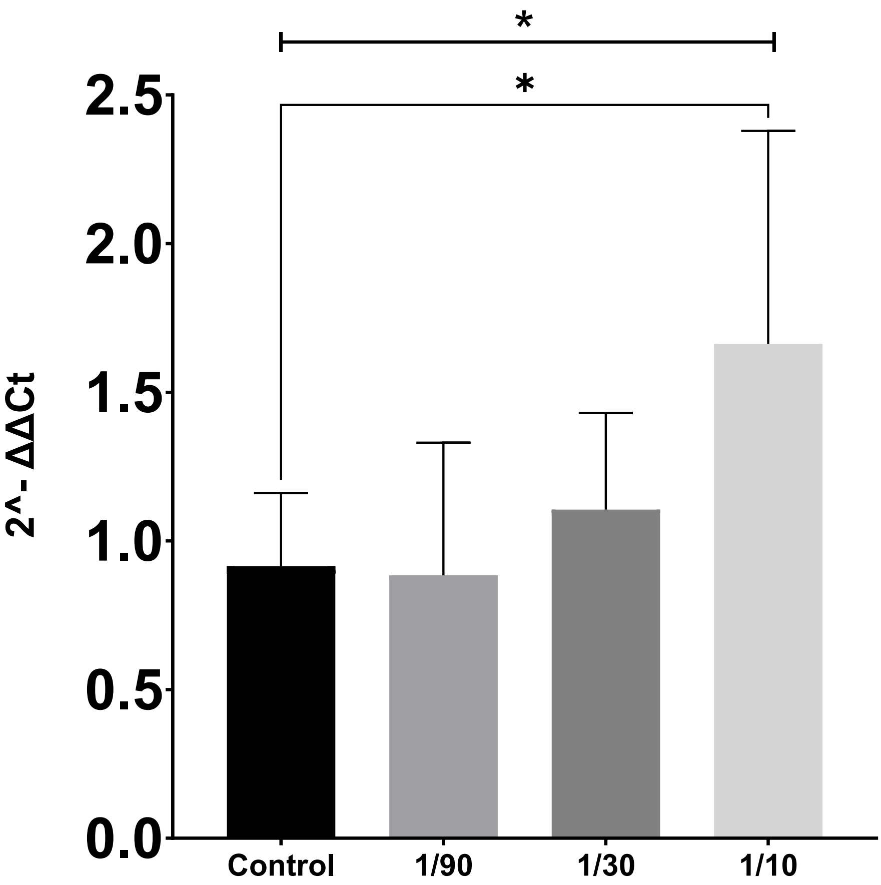

Supplementary Figure 2. Dose–response analysis of exosome treatment on fibroblasts (COL1A2 expression). Exosomes from a single isolation lot (1.5×10^9 particles/mL) were applied at 1/90, 1/30, and 1/10 dilutions; untreated cells served as control. Relative COL1A2 expression was quantified by the $2^{-}\Delta\Delta Ct$ method. Points show individual biological replicates; bars indicate the median with interquartile ranges (IQR). Kruskal–Wallis test across groups showed a trend toward significance ($P = 0.078$). Pairwise Mann–Whitney U tests (two-tailed, exact) identified a significant increase for control vs 1/10 ($P = 0.0185$), whereas control vs 1/30 and control vs 1/90 were not significant ($P > 0.05$). In the graphic, bold horizontal bars denote the Kruskal–Wallis omnibus result and thin horizontal bars denote Mann–Whitney comparisons. Significance symbols: *$P < 0.05$.
