# Supplementary material for: Cryopreserved exosomes derived from hypoxic Wharton’s jelly mesenchymal stromal cells enhance fibroblast proliferation, upregulate COL1A2 expression, and mitigate senescence
Source: Front Med (Lausanne). 2025 Dec 17;12:1692585. doi: 10.3389/fmed.2025.1692585 (PMC12753964; doi:10.3389/fmed.2025.1692585)
Supplement: Supplementary file 6 [file Image_3.pdf]

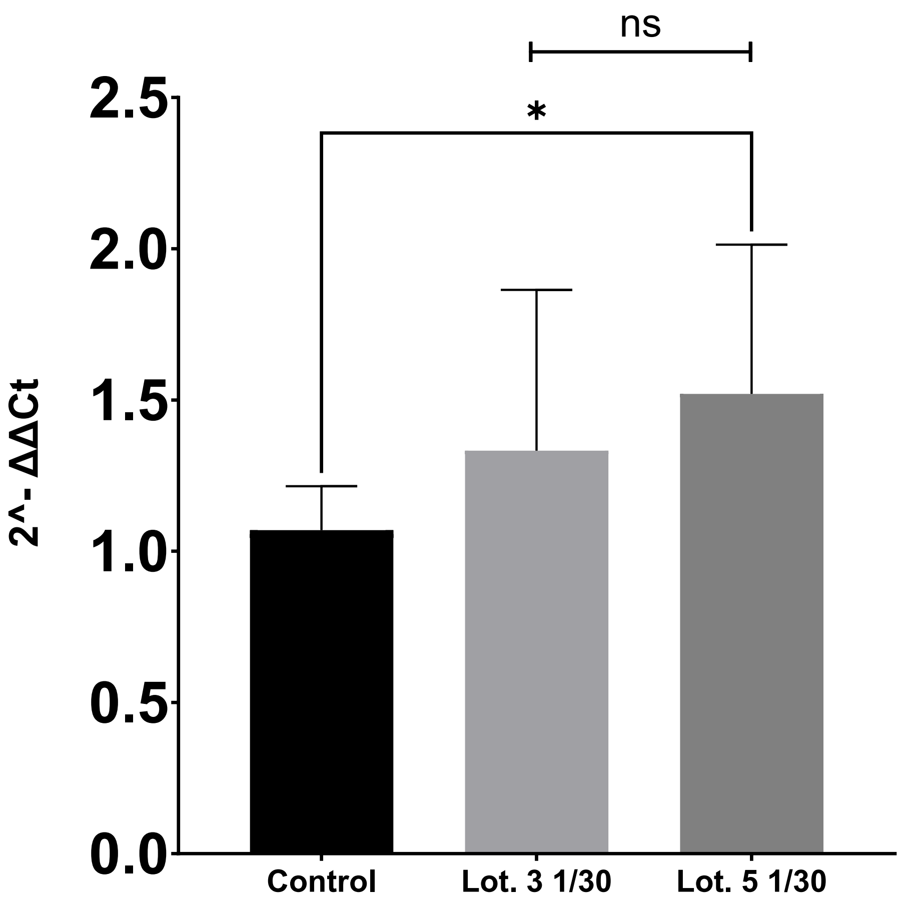

Supplementary Figure 3. Lot-to-lot comparison of exosome treatment on fibroblasts (COL1A2 expression). Cryopreserved, hypoxia-conditioned WJ-MSC exosomes from two independent lots (Lot 3 and Lot 5; stock 1.5×10^9 particles/mL) were applied to fibroblasts at a 1/30 dilution; untreated cells served as control. Relative COL1A2 expression was quantified by qPCR using the $2^{-}\Delta\Delta Ct$ method (reference gene: PUM1). Technical replicates were averaged to yield a single value per biological replicate ($n = 3$ independent experiments per condition). Both lots showed higher median COL1A2 expression than control. Pairwise Mann–Whitney U tests (two-tailed, exact) indicated a significant increase for Lot 5 (1/30) vs control ($P < 0.05$), no significant difference for Lot 3 (1/30) vs control, and no significant difference between Lot 3 vs Lot 5. Bars display the median with interquartile range (IQR); "ns" denotes not significant.
